# Brain Tumor Causing Atrial Fibrillation in an Otherwise Healthy Patient

**DOI:** 10.7759/cureus.1601

**Published:** 2017-08-23

**Authors:** Nilesh H Pawar, Farhad F Vasanwala, Melvin Chua

**Affiliations:** 1 Department of General Medicine, Sengkang General Hospital, Singhealth, Singapore

**Keywords:** brain tumor, brain neoplasms, atrial fibrillation, brain-heart connection, neuro-oncology

## Abstract

We report a case of a 57-year-old man who presented with an episode of syncope resulting in an accident. On presentation, he was found to have atrial fibrillation (AF). A brain imaging done to exclude intracranial hemorrhage revealed a brain tumor instead. Recently, AF has been used as a marker for occult cancer. Thus, we hypothesize that AF in our patient was a result of the existing brain tumor and not simply a coincidence. AF may help in diagnosing brain tumors in asymptomatic or oligosymptomatic patients at an early stage, decreasing mortality and morbidity significantly.

## Introduction

Brain tumors can present with a headache, a seizure, or other neurological symptoms, depending on the location of the tumor [1-3]. They are usually diagnosed during the stages when the tumor has progressed and becomes symptomatic [2-3]. During the early phase, brain tumors are often asymptomatic or oligosymptomatic, where diagnoses have only been made incidentally in patients with no history of neurological problems [2-3].

Atrial fibrillation (AF) is the most common type of cardiac arrhythmia [4]. The most common causes of AF are hypertensive heart disease, coronary heart disease, diabetes, hyperthyroidism, smoking, and alcohol abuse [4-5]. Recent studies have shown that it can be used as a marker for occult cancer [4,6] and may also help in diagnosing patients with asymptomatic or oligosymptomatic brain tumors. Here, we present an interesting case of a patient with no risk factors for AF, and the cause of his new AF was likely his brain tumor.

## Case presentation

A 57-year-old, right-handed man presented with mild cellulitis over the dorsum of his left lower limb. He was involved in a road traffic accident a week prior to his admission, resulting in an abrasion over the dorsum of his left foot. On further questioning, the patient admitted to an episode of syncope while riding his bike, which caused him to hit a car at the traffic light. He had no prodrome and made full recovery after a brief loss of consciousness, lasting for 2-3 minutes. He did not complain of any other alarming symptoms prior to or after his accident. He did not have any significant past medical history or family history of note.

**Figure 1:**
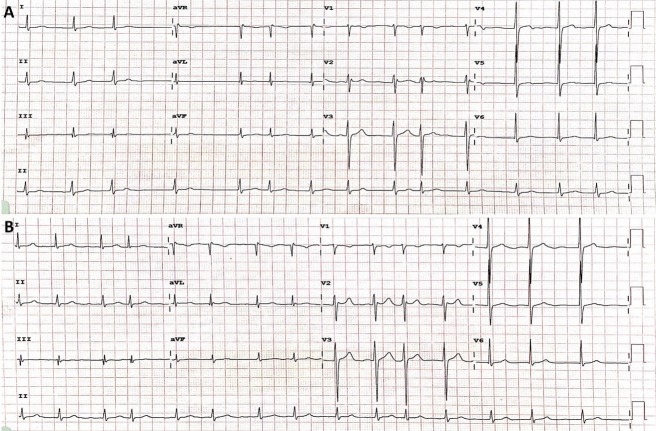
Electrocardiograms: (A) on admission and (B) 3 hours later, showing persistent atrial fibrillation.

On examination, he was apyrexial and his blood pressure was 148/96 mm Hg, but this settled subsequently to less than 130/80 mm Hg. There were no focal neurological findings. However, his heart rate was irregularly irregular and his electrocardiograms (Figure 1A, 1B) revealed AF, which was confirmed on a 24-hour Holter monitor. Echocardiography demonstrated normal valve morphology and no other abnormality was revealed.

**Figure 2:**
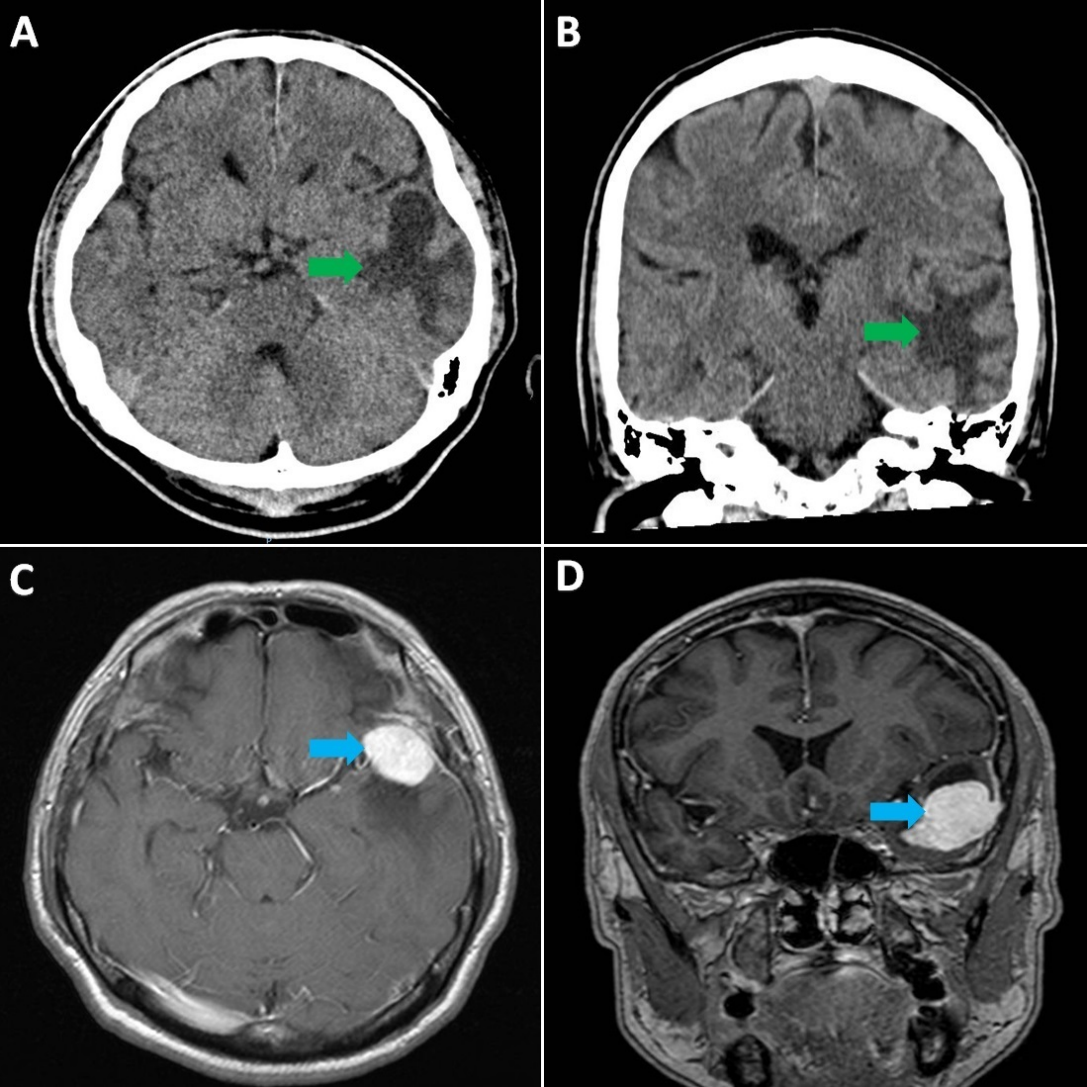
CT scan of head: axial (A) and coronal (B] views, showing suspected mass lesion (green arrows) in the left temporal lobe with surrounding vasogenic oedema and mass effect; MRI of brain: axial [C] and coronal [D] views, showing a well-defined, avidly enhancing extra-axial mass lesion (blue arrows) in the left, middle cranial fossa. CT = computed tomography MRI = magnetic resonance imaging

All his laboratory tests were normal, including electrolytes, renal, liver, and thyroid function. Complete blood counts and inflammatory markers were within normal limits, with a slight elevation of the C-reactive protein (CRP) of 13.3 mg/L (reference range, 0.2-9.1 mg/L). A computed tomography (CT) scan of the head (Figure 2A, 2B) demonstrated a suspected mass lesion centered in the left temporal lobe. Magnetic resonance imaging (MRI) (Figure 2C, 2D) of the brain confirmed the presence of a well-defined, avidly enhancing extra-axial mass lesion over the left temporal lobe. This patient’s care was transferred to the neurosurgery department for further management.

## Discussion

Syncope is a transient and self-limited loss of consciousness as a consequence of transient cerebral hypoperfusion [7]. Persistent AF can cause syncope by hemodynamic dysfunction, causing a decrease in cardiac output and leading to cerebral hypoperfusion [7]. Recent studies have shown that patients with new-onset AF have a reasonably higher risk of developing cancer within the next three months, with these risks increasing after three months [4].

Our patient did not have any of the risk factors for AF, as described in the literature [5], making it a possibility that there is an association between the AF and the patient’s brain tumor. Bacterial infections with a substantially high CRP can cause AF [8]. However, our patient only had very mild cellulitis with a CRP of 13.3 mg/L, making it less likely to be the cause of his AF. The cases of brain tumors associated with AF described in the literature thus far demonstrate that AF is usually present during the aggressive phase of the disease [8]. The mechanism proposed in these cases is the compression of the heart by intra-thoracic masses due to intra-thoracic metastases from the aggressive brain tumor, giving rise to AF [9]. However, our patient developed AF during the early stages of the disease with no evidence of metastases. Therefore, we hypothesize that a brain tumor can cause AF even in the absence of metastases. Neuroanatomic connections exist between the brain and the heart, activation of which results in brain-related arrhythmias [10]. Neural inputs are supplied to the heart via parasympathetic ganglia and the intermediolateral gray columns of the spinal cord, which innervates the crucial autonomic structures of the heart and affects its rate and rhythm [10]. The release of catecholamines from the nervous system leads to cardiac damage and induces arrhythmias [10]. Brain tumors have been associated with AF, bradycardia, and cardiac syncope [9-10], suggesting an association of brain tumors with the heart’s rate and rhythm, favoring our hypothesis.

Thus, as brain tumors are usually asymptomatic or oligosymptomatic in the early stages, it makes diagnosis difficult [2]. If brain tumors are discovered at an early stage, rapidly enlarging brain tumors can be ruled out by surveillance scans [3] and can considerably decrease morbidity and mortality by early intervention. It is, therefore, essential that all physicians should be made aware of AF as a potential indicator of brain tumors and begin screening for brain tumors in all patients with new-onset AF. 

## Conclusions

This report demonstrates that brain tumors can cause AF in the early stages, even in the absence of metastasis. Neuroanatomic connections exist between the nervous and cardiovascular systems, and by releasing catecholamines, the nervous system may induce arrhythmias, including AF. AF may help in diagnosing brain tumors and could be potentially used as a marker of occult brain tumors. Early intervention may help to reduce mortality and morbidity considerably in this group of patients.
